# Development, implementation and evaluation of a podiatry led “high risk foot” student clinic at the Queensland University of Technology

**DOI:** 10.1186/1757-1146-4-S1-P9

**Published:** 2011-05-20

**Authors:** Damien Clark, Lloyd Reed, Ewan M Kinnear, Peter A Lazzarini

**Affiliations:** 1 School of Public Health, Queensland University of Technology, Brisbane, Australia; 2 Department of Podiatry, Metro North Health Service District, Queensland Health, Brisbane, Australia; 3 Allied Health Research Collaborative, Metro North Health Service District, Queensland Health, Brisbane, Australia; 4 Institute of Health and Biomedical Innovation, Queensland University of Technology, Brisbane, Australia

## Background

Diabetic foot complications are acknowledged as the leading cause of amputation and diabetes-related hospitalisation. Podiatry-led multi-disciplinary clinics are recognised as important strategies to improve diabetic foot outcomes. Data suggests diabetic foot complications are increasing at a faster rate than diabetes is being diagnosed. A reliable, competent workforce is urgently required to adequately manage patients with diabetes foot complications. Clinical training is known to have a beneficial impact on diabetic foot ulcer outcomes. As the basis for podiatry clinical training occurs at the undergraduate level in Australia, the development of students equipped with best practice skills to manage the growing population of diabetic foot complications is essential. The aim of this paper is to develop, implement and evaluate a student led “high risk foot” clinic and evaluate its impact on undergraduate learning and diabetic foot outcomes.

## Methods

In January 2011 a designated Queensland University of Technology (QUT)/ Queensland Health (QH) “high risk foot” podiatry led student clinic will commence on the QUT campus with support from the QUT/QH clinical educator and other senior podiatrists. To complement the student clinical experience, placements in QH “high risk” foot services and exposure to simulation training in high risk foot management will be fostered. Primary evaluation will be undertaken quarterly and involve addressing satisfaction and clinical competencies. Surveys will be obtained from students, clinical supervisors and placement supervisors. Further evaluation of students’ diabetic foot clinical indicators and patient outcomes will also be evaluated.

## Results

The presentation will review the initial planning and implementation of the “high risk foot” student clinic at QUT. The first three month evaluation will be presented at the conference.

## Conclusions

The explosion of diabetic foot complications requires a well skilled workforce. Undergraduate education remains at the core of podiatry learning in Australia. The development of student “high risk foot” clinics may be an innovative and effective strategy to meet the needs of Australia’s diabetic foot complications. It is envisaged two years worth of data will be analysed and presented to the 2013 Australasian Podiatry Conference.

